# Editorial: Case Reports in Visceral Surgery: Tumors of the Liver

**DOI:** 10.3389/fsurg.2022.841783

**Published:** 2022-01-27

**Authors:** Gabriel Sandblom

**Affiliations:** ^1^Department of Surgery, Karolinska Institutet (KI), Solna, Sweden; ^2^Department of Surgery, Stockholm South General Hospital, Stockholm, Sweden

**Keywords:** associating liver partition and portal vein ligation for staged hepatectomy, incidentaloma, leiomyoma, robot-assisted surgery, near-infrared fluorescence, hepatic adenoma, neuroendocrine neoplasia

Surgical resection remains the mainstay of curative treatment for primary hepatic malignancies and colorectal metastases to the liver. Most experience regarding liver surgery has been gained from resection of colorectal metastases, cholangiocarcinoma, and hepatocellular cancer (HCC). Outcome after liver surgery has improved over recent decades. However, there are many clinical challenges in the preoperative assessment and management of liver tumors that entail novel solutions that have yet to be evaluated, especially rare tumor forms.

The development of imaging techniques and extensive use of computer tomography has resulted in a growing number of small liver lesions being detected. Many of these are benign and do not require surgical treatment, but may sometimes be difficult to distinguish from hepatocellular carcinoma. HCC is the most common primary liver cancer. There are, however, rare cases where the tumor is a combination of HCC and neuroendocrine tumor (NET), possibly a result of poorly differentiated tumor cells developing into different lines such as NET and HCC (Lan et al.).

Liver lesions may be resected to reduce local symptoms as long as they are carefully selected and a benign diagnosis has been confirmed preoperatively. The histopathologic complexity of the liver and its involvement in various functions of the digestive and immunological systems make it a hotbed for tumors of various origins. Gonadal hormone substitution may induce hepatic adenomas that vanish as soon as treatment is interrupted (Liu et al.). Leiomyomas are mesenchymal tumors that are sometimes seen in immunosuppressed patients (Djokic et al.). The choice between surgical treatment and conservative management thus requires careful consideration of the specific characteristics of each tumor, preferably in multidisciplinary collaboration.

Surgical methods have developed rapidly over the last two decades, in particular robot-assisted laparoscopy. This has made it possible to carry out minimally invasive surgery that was considered impossible only a few years ago, including resections along major vessels and deeply located lesions (Solomonov et al.). Although there are few well-designed randomized controlled trials confirming the safety and cost-benefit of robot-assisted liver surgery, rapid development of the technique may render the latest presented report outdated as soon as it is published.

The ability of the liver parenchyma to regenerate has been used as a method to reduce the risk for postoperative organ failure. The combination of liver partition and local portal vein ligation for staged hepatectomy (ALPPS) induces hypertrophy of the liver remnant, and has been shown to be a way of enabling more extensive liver resection. ALPPS is carried out as a two-stage procedure. In the first stage, transection along the part of the liver to be resected is made together with local portal ligation. The patient is then allowed to recover for approximately 2 weeks during which the liver remnant regenerates. In the second stage, the deportalized liver lobe is removed. In cases with cirrhosis, however, the liver remnant may fail to regenerate. A potential solution in such cases may be hepatic arterial infusion chemotherapy to the part of the liver to be resected (Zhuo et al.). The technique has been tested and results are promising. However, this technique should be evaluated further before being accepted in routine clinical practice.

Visualization of bile ducts and tissue planes is a great challenge in liver surgery. Near-infrared fluorescence (NIRF) imaging after intravenous injection of indocyanine green (ICG) is a method that is gradually becoming routine in benign gallbladder surgery. A recent report showed that NIRF can also be used in surgery for gallbladder cancer, enabling wedge liver resection even when the gallbladder cancer invades the parenchyma along the bile ducts (Yu et al.). NIRF will probably become a routine technique in various situations, particularly when detecting ischaemia and visualizing the bile ducts intraoperatively.

Some liver tumors are today considered inaccessible for surgery with radical intent, but with continuing development of techniques for managing various liver lesions, we anticipate new progress in the near future. New imaging techniques that incorporate artificial intelligence may help in preoperative planning and execution of surgery of these cancer forms, further extending the horizon of liver tumor management. This, together with the development of robot-assisted laparoscopy, will lead to minimally invasive surgery becoming the first-hand choice in liver surgery.

## Author Contributions

The author confirms being the sole contributor of this work and has approved it for publication.

## Conflict of Interest

The author declares that the research was conducted in the absence of any commercial or financial relationships that could be construed as a potential conflict of interest.

## Publisher's Note

All claims expressed in this article are solely those of the authors and do not necessarily represent those of their affiliated organizations, or those of the publisher, the editors and the reviewers. Any product that may be evaluated in this article, or claim that may be made by its manufacturer, is not guaranteed or endorsed by the publisher.

